# LPG Dependence after a Suicide Attempt

**DOI:** 10.1155/2015/643253

**Published:** 2015-01-13

**Authors:** Ebru Aldemir, Betül Akyel, A. Ender Altıntoprak, Rezzan Aydın, Hakan Coşkunol

**Affiliations:** ^1^Institute on Drug Abuse, Toxicology and Pharmaceutical Sciences, Ege University, 35040 İzmir, Turkey; ^2^Department of Psychiatry, Ege University, 35040 İzmir, Turkey; ^3^Department of Child and Adolescent Psychiatry, Ege University, 35040 İzmir, Turkey

## Abstract

Inhalant abuse is a problem that is getting more common all around the world. The increase in prevalence of inhalant abuse escalates morbidity and mortality rates. About 22% of people using inhalant have died at their first attempt. Particularly propane, butane, or propane-butane mixture has highest mortality rates. Sudden sniffing death syndrome, cardiomyopathy, central nervous system toxicity, hematological abnormalities, kidney toxicity, and hepatocellular toxicities are the major complications of inhalant abuse. Herein we present a patient with inhalant use disorder. At the age of 19, after a stressful life event he had unsuccessfully tried to suicide by inhaling LPG (liquefied petroleum gas, a mixture of butane and propane gases). After he realized that he had hallucinations and felt better during the inhalation, he started to abuse it. He was addicted to LPG for 10 years at the time of admission. Besides being dangerous for the society security, this intense level of LPG inhalation (12 liters a day) not giving any physical harm makes this case interesting.

## 1. Introduction

Inhalant abuse has been reported from most of the world, mainly among adolescents. Inhalants are preferred because these substances are easy to access and cheap to purchase and have rapid effects [1].

There are various inhalants with different ingredients. McGarvey et al. [2] have classified abused inhalants, which are used for various purposes, into different subgroups:

1. medical anesthetic gases: nitrous oxide, helium, and oxygen (compressed air);
2. industrial/household solvents: bleach, glue, hair dye, nail polish, nail polish remover, paint, paint thinner, and shoe polish;
3. art/office solvents: permanent markers, correction fluids;
4. gases used in nonmedical products: gasoline, propane, kerosene, butane, lighter fluid, antifreeze, and freon;
5. household aerosol propellants: hair spray, spray paint, deodorant, and air freshener;
6. aliphatic nitrites (poppers): asthma inhalers.

The prevalence of abuse of these groups of inhalants differs by country. It has been reported that the most commonly abused inhalants in Turkey are paint thinners and adhesives [3].

Inhalant abuse has increased; hence mortality and morbidity rates have also increased. Sudden death can occur even on the first experience of abuse. It was determined that approximately 22% of the people who used inhalants died during their first use [4]. Sudden deaths are usually caused by abuse of butane, propane, or butane/propane mixture [5–7].

LPG (liquefied petroleum gas) is a mixture of gases used as a fuel in heating, cooking, and lighting. LPG is colorless and odorless and it is prepared by refining petroleum or natural gas. It is a mixture of butane and propane gases (approximate ratio is 70 : 30 by mass in Turkey) and can be stored in 12-liter cylinder tanks. At room temperature LPG is a gas, but it can easily be compressed to a liquid form which is easier to store and transport [8].

Here we present a case of a 31-year-old man who tried to commit suicide by sniffing LPG ten years ago. He reported that he had visual hallucinations and felt euphoric during the sniffing and afterwards he became addicted to LPG.

## 2. Case Report

Mr. Y. is 31-year-old, high school graduate, single, and works in a factory as a chef. He was referred to Ege University Psychiatry Department outpatient unit in January 2014 in order to overcome his LPG dependence. He first used LPG, when he was 19 years old, to commit suicide. The reason of the suicide attempt was his girlfriend's family's refusal regarding their marriage. During this attempt, he realized that he had hallucination of his girlfriend and could also talk to her, which made him feel better. He also recognized that LPG made him feel elated. After the suicide attempt his relatives found him unconscious and they rescued the patient. Thereafter, he began to abuse LPG anytime when he was anxious. The amount and frequency of usage increased over time, and he would eventually consume 12 liters at once. Although he had previously used LPG outdoors so as not to harm other people, in time, he began using it indoors. He reports that he feels better and more self-confident, and he talks to whomever he wants while inhaling LPG. The patient indicates that effects of LPG have lasted for five to ten minutes. He joined the army when he was 20 years old. When he was in the army, he consumed LPG less than before, but still continued. After the army, he began to work in a factory as a worker. During that time, he reported willingly remaining abstinent from LPG for two years without any professional help. At the end of those two years, he started to use LPG again as his responsibilities increased. The frequency of use increased when his brother committed suicide by hanging himself four years ago. He had used LPG for two months without leaving his home because he thought that he was responsible for his brother's death by not being with him. He experienced a loss of appetite and subsequent weight loss. He had headaches and bone pain. He had a visual hallucination once in which his brother told him to get rid of his addiction, whereupon he decided to be treated. He was hospitalized and got treatment 3 years ago in an Alcohol and Drug Research, Treatment and Training Center for 20 days. After discharge, he used some medications for two months, but he could not remember their names. He did not have any complaints thereafter. He decided to marry but then he had concerns about financial problems. As a result of these concerns, he began to abuse LPG again. When his distress and tension were increased, he was inhaling LPG until he finished the whole 12 liters of it at once or until his friends came to take it away from him. He could not work when he was abusing LPG. His boss and coworkers were aware of his addiction to inhalants. He was referred to our outpatient unit after his girlfriend broke up with him when she had learned he was addicted to LPG.

In the psychiatric examination of the patient, insomnia, depressive mood, anxiety, unsuccessful efforts to cut down inhalant use, continued inhalant use despite harmful consequences, craving for inhalant, breakdown in his interpersonal communication, using inhalant even in dangerous conditions, occupational problems because of inhalant use, and tolerance to inhalant were observed. His brother had committed suicide by hanging himself and his aunt had a psychotic disorder. He stated that he did not have any medical disorders. Individual and community risks (sudden death and explosion) were considered, and he was hospitalized at Ege University Alcohol and Drug Research and Treatment Center. He was diagnosed with both “Inhalant Use Disorder, Severe, and Major Depressive Disorder” according to DSM-5 (Diagnostic and Statistical Manual of Mental Disorders). There were no abnormalities on the Mini Mental State Examination and neurological examination. There were no pathological cranial magnetic resonance or diffusion magnetic resonance imaging (MRI) findings. There were also no abnormalities in the liver, kidney, and thyroid function tests and no electrolyte imbalance or complete blood count abnormalities. Any pathology was not determined by Wechsler Memory Scale subtests, Stroop, and verbal fluency tests regarding memory, attention, and executive functions. Additionally, he underwent cardiologic, pulmonary, and internal medicine examinations for the medical problems which may arise due to chronic LPG abuse. Although no pathology was determined for the pulmonary diseases, it had been shown that acute respiratory distress syndrome, shortness of breath, or pneumonia may occur if LPG is inhaled continuously. Cardiologic evaluation reported that he had dyspnea on effort; however, there were no pathological findings on echocardiography or Holter electrocardiography. There were no problems in terms of internal medicine. Health parameters of patient and expected toxic consequences of inhalant abuse on these parameters have been shown in Table 1. He began taking paroxetine 20 mg/day for depressive symptoms and quetiapine 100 mg/day for its anxiolytic and sedative effects. Resolution of depressive symptoms was observed on the tenth day of treatment, and he participated in relapse prevention groups. A marked resolution in depressive symptoms was seen at the end of the third week, and he stated that he did not have craving for LPG anymore. He was discharged from inpatient unit and admitted to outpatient unit. He had not any complaints in follow-up psychiatric evaluations and the dose of quetiapine was gradually terminated. He has been abstinent for three months.

## 3. Discussion

In this paper, we report a patient who attempted suicide by sniffing LPG 10 years ago. While sniffing he realized that it makes him feel euphoric and he had hallucinations, and he therefore became addicted to LPG.

Mortality and morbidity increase as the frequency of use of inhalants increases. But “Sudden Sniffing Death Syndrome” is not related to frequency of use. It is the leading cause of mortality related to inhalant abuse and can occur even during the first experiment. Sudden Sniffing Death Syndrome caused by cardiac arrhythmia can occur during or after inhalation [9]. The next leading causes of acute death attributed to inhalant use are suffocation, aspiration, and accidental injury. Fortunately, there were no such complications in our patient despite of long term (10 years) and intense abuse. Finally, deaths due to chronic use of inhalants are attributed to cardiomyopathy, myocardial infarction, central nervous system toxicity (dementia, brain stem disorder), hematologic abnormalities (aplastic anemia, leukemia), kidney toxicity (nephritis, nephrosis, and tubular necrosis), and hepatocellular carcinoma [9, 10]. There were no abnormalities on cardiac, cranial, internal, and thoracic examinations in our patient.

According to a study on LPG workers, it was determined that red blood cell count, hemoglobin level, platelet count, kidney function tests (urea, uric acid, and creatinine), and liver function tests (AST, ALT) are all significantly higher in the LPG group compared to the control group. It was found that self-reported health-related complaints among LPG workers were higher compared to healthy controls [11]. Fortunately, there were no pathologic findings in the hematologic and biochemical tests of our patient.

Muscle weakness, tremor, peripheral neuropathy, cerebellar dysfunction, chronic encephalopathy, and dementia have been described after chronic inhalant abuse [9]. White matter degeneration and abnormalities in thalamus, basal ganglia, pons, and cerebellum in cranial MRI are generally seen in these patients [12]. Memory, attention, problem solving, learning, and visual-motor coordination are also affected [13, 14]. It is surprising that we did not see any pathological findings on the Mini Mental State Examination, neurological examination, neurocognitive tests, cranial MRI, and diffusion MRI of our patient.

## 4. Conclusion

The gas mixture of propane-butane is among the most lethal inhalant substances. These gases also cause severe complications in terms of physical health. In this paper, a patient who has been very severely addicted to inhalant propane-butane for ten years is reported. He did not have any accidents, although he had been using very large amounts of inhalant substances in closed environments, which is a potential threat to society. No physical or neurocognitive pathologies were determined in comprehensive investigations which make this case interesting.

## Figures and Tables

**Table 1:** Health parameters of patient and expected toxic consequences of inhalant abuse on these parameters.

Health parameters (reference range)	Parameters of patient	Expected toxic consequences of inhalant abuse
Complete blood count		
Red blood cell (4.3–5.7 × 10^6^ *μ*L)	5.04	High red blood cell count
White blood cell (4.5–11.0 × 10^3^ *μ*L)	7.41	High hemoglobin level
Platelet (150–450 × 10^3^ *μ*L)	307	Aplastic anemia
Hemoglobin (13.2–17.3 g/dL)	15	Leukemia
Hematocrit (39–49%)	44.2	
Liver function tests		
Aspartate transaminase (<35 U/L)	18	High levels of transaminases
Alanine transaminase (<45 U/L)	24	
Gamma-glutamyl transferase (<55 U/L)	31	
Total protein (6.4–8.3 g/dL)	6.7	
Albumin (3.5–5.2 g/dL)	4.5	
Kidney function tests		
Urea (10–50 mg/dL)	31	High levels of kidney function tests
Uric acid (3.5–7.2 mg/dL)	4.9	Nephritis
Creatinine (0.7–1.3 mg/dL)	0.93	Nephrosis
		Tubular necrosis
Cranial and cranial diffusion MRI^*^	Normal MRI findings, right maxillary sinus retention cyst	White matter degeneration Abnormalities in thalamus, basal ganglia, pons, and cerebellum
Neurocognitive tests (age and education adjusted reference scores)		
MMSE score^**^ (>23)	30	Dementia
Wechsler Memory Scale subtests		Cognitive deficits on memory, attention, problem solving, learning, and visual-motor coordination
Digit-span forward (>4,72 ∓ 1,46)	6	
Digit-span backward (>4,97 ∓ 1,53)	5	
Stroop test (max. 50 seconds)	21	
Verbal fluency test (40 ∓ 12)	40	
Cardiologic evaluation		
Electrocardiography (ECG)	Normal sinus rhythm with the heart rate of 89/minute	Sudden sniffing death syndrome Cardiomyopathy Arrhythmias Myocardial infarction
Holter ECG	Rare ventricular and supraventricular extrasystoles	
Echocardiography	Normal echocardiographic findings	

^*^Magnetic resonance imaging. ^**^Mini Mental State Examination.
